# Effect of low-level laser therapy on tooth-related pain and somatosensory function evoked by orthodontic treatment

**DOI:** 10.1038/s41368-018-0023-0

**Published:** 2018-07-02

**Authors:** Song Wu, Yinan Chen, Jinglu Zhang, Wenjing Chen, Sheng Shao, Huijie Shen, Ling Zhu, Ping Ye, Peter Svensson, Kelun Wang

**Affiliations:** 1 Orofacial Pain and TMD Research Unit, Institute of Stomatology, Affiliated Hospital of Stomatology, Nanjing Medical University, 136 Hanzhong Road, Nanjing, China; 2 Institute of Stomatology and Department of Orthodontics, Affiliated Hospital of Stomatology, Nanjing Medical University, 136 Hanzhong Road, Nanjing, China; 3 Department of Stomatology, The Children’s Hospital, Zhejiang University School of Medicine, Hangzhou, China; 4 Institute of Dental Research, Central for Oral Health, Westmead Hospital, Westmead, Australia; 5 Section of Orofacial Pain and Jaw Function, Department of Dentistry and Oral Health, Aarhus University, Aarhus, Denmark; 6 Department of Dental Medicine, Karolinska Institutet, Huddinge, Sweden; 7 Scandinavian Center for Orofacial Neurosciences (SCON), Stockholm, Sweden; 8 Center for Sensory-Motor Interaction (SMI), Department of Health Science and Technology, Aalborg University, Aalborg, Denmark

## Abstract

Low-level laser therapy (LLLT) may have an effect on the pain associated with orthodontic treatment. The aim of this study was to evaluate the effect of LLLT on pain and somatosensory sensitization induced by orthodontic treatment. Forty individuals (12–33 years old; mean ± standard deviations: 20.8 ± 5.9 years) scheduled to receive orthodontic treatment were randomly divided into a laser group (LG) or a placebo group (PG) (1:1). The LG received LLLT (810-nm gallium-aluminium-arsenic diode laser in continuous mode with the power set at 400 mW, 2 J·cm^–2^) at 0 h, 2 h, 24 h, 4 d, and 7 d after treatment, and the PG received inactive treatment at the same time points. In both groups, the non-treated side served as a control. A numerical rating scale (NRS) of pain, pressure pain thresholds (PPTs), cold detection thresholds (CDTs), warmth detection thresholds (WDTs), cold pain thresholds (CPTs), and heat pain thresholds (HPTs) were tested on both sides at the gingiva and canine tooth and on the hand. The data were analysed by a repeated measures analysis of variance (ANOVA). The NRS pain scores were significantly lower in the LG group (*P* = 0.01). The CDTs, CPTs, WDTs, HPTs, and PPTs at the gingiva and the PPTs at the canine tooth were significantly less sensitive on the treatment side of the LG compared with that of the PG (*P* < 0.033). The parameters tested also showed significantly less sensitivity on the non-treatment side of the LG compared to that of the PG (*P* < 0.043). There were no differences between the groups for any quantitative sensory testing (QST) measures of the hand. The application of LLLT appears to reduce the pain and sensitivity of the tooth and gingiva associated with orthodontic treatment and may have contralateral effects within the trigeminal system but no generalized QST effects. Thus, the present study indicated a significant analgesia effect of LLLT application during orthodontic treatment. Further clinical applications are suggested.

## Orthodontics: Laser therapy potential to reduce pain

Repeated applications of low-level laser therapy during and after orthodontic procedures could help reduce pain and discomfort. Many orthodontic patients experience pain in the hours and days following treatment, which impacts on treatment participation. Wenjing Chen at Nanjing Medical University and co-workers assessed whether low-level laser therapy (LLLT) can reduce pain and sensitivity in teeth and surrounding tissues. They divided 40 patients into two groups; one was given repeated LLLT in the hours and days following procedures, while the other group received a placebo course. They tested both groups for sensitivity to stimuli including heat and pressure, in the mouth and on the hands. LLLT significantly reduced pain in the mouth relative to the placebo group. No differences were found in tests on the hands, suggesting LLLT works as a targeted analgesia.

## Introduction

The most common problems for patients during orthodontic treatment are pain and discomfort evoked by the appliances and mechanical loading.^1–6^ It has been reported that ~90% of orthodontic patients will experience pain during orthodontic treatment.^7^ Pain associated with dental treatment in general is strongly influenced by factors, such as personality, gender, and, especially, and previous dental experience.^2,8,9^ Orthodontic treatment has the goal of aligning the dental occlusion by moving the teeth with consistent mechanical forces applied to the teeth. The loading and forces may lead to an inflammatory process in the periodontal ligament, which can cause pain.^10^ The pain usually begins within 4 h after the force is applied, reaches a peak after ~24 h, and then dissipates by day 7.^11,12^ The intensity of the pain during orthodontic treatment is sometimes reported to be even stronger than the pain related to dental extractions.^13^ Thereby, it could be one of the reasons for non-compliance with and even withdrawal from orthodontic treatment.

Low-level laser therapy (LLLT) has attracted attention for decades because of its obvious advantage in pain management as a non-invasive and inexpensive technique without significant adverse effects.^4,14,15^ Overall, studies on clinical efficacy have shown equivocal results.^16^ The efficacy of LLLT in alleviating orthodontic pain has also been studied in recent years.^14,17–22^ It has been reported that LLLT has analgesic properties and anti-inflammatory effects^23–25^ through increases in the local blood flow, reduction of prostaglandin levels E2 and inhibition of cycloxygenase-2.^13,24^ Various studies have been designed to investigate the pain during orthodontic treatment, including telephone interviews and questionnaires.^1,3,26,27^ However, because of small sample sizes, controversial results, and different methodological issues in previous studies, good evidence for a positive treatment effect of LLLT is still lacking. Randomized, placebo-controlled and double-blinded studies are needed.^14,16,28^

Quantitative sensory testing (QST) is a psychophysical method in which different standardized stimulus modalities (e.g., thermal and mechanical) are applied to different tissues (skin, joint, mucosa, and muscles), and the test person’s response, in terms of a sensory or pain threshold or a report of the magnitude of the perceived intensity, is assessed.^29^ QST in the trigeminal region has been characterized in terms of specificity, sensitivity, repeatability, and reliability.^30,31^ The application of QST could help to study the underlying neurobiological mechanisms of orthodontic pain and could serve as a valuable measure to determine the effects of LLLT on somatosensory function. One of our recent studies has shown that pressure pain thresholds (PPTs) applied to the teeth have excellent intra-examiner and inter-examiner agreements in healthy participants.^32^ A modified intra-oral QST has been applied in several of our previous studies^32–34^ and has been shown to be an easy and reliable technique for assessing mechanical pain sensitivity (e.g., mechanical allodynia and hyperalgesia) in the periodontal ligament, which is associated with endodontic or periodontal conditions.

As a measure of self-reported levels of pain, the numerical rating scale (NRS) score, in which 0 represents “no pain” and 10 indicates “the most pain imaginable”, can be recorded from the participants. The aim of the present study was to evaluate the effect of LLLT on the self-reported pain and somatosensory function induced by orthodontic treatment using our recently established intra-oral QST techniques in a randomized, placebo-controlled and double-blinded study design.

## Results

### Baseline differences

There were no significant age or gender differences between the laser group (LG) and placebo group (PG) at baseline (*P* > 0.916). There were no significant differences between the LG and PG at baseline for cold detection threshold (CDT) (ANOVA; *F* = 1.57, df = 1, *P* = 0.215), cold pain threshold (CPT) (ANOVA; *F* = 0.26, df = 1, *P* = 0.223), warmth detection threshold (WDT) (ANOVA; *F* = 0.28, df = 1, *P* = 0.597), heat pain threshold (HPT) (ANOVA; *F* = 0.13, df = 1, *P* = 0.721), pressure pain threshold (PPT) at the gingiva (ANOVA; *F* = 1.03, df = 1, *P* = 0.315), or pressure pain threshold (PPT) at the canine tooth (ANOVA; *F* = 0.916, df = 1, *P* = 0.347).

### NRS pain scores

**Fig. 1:**
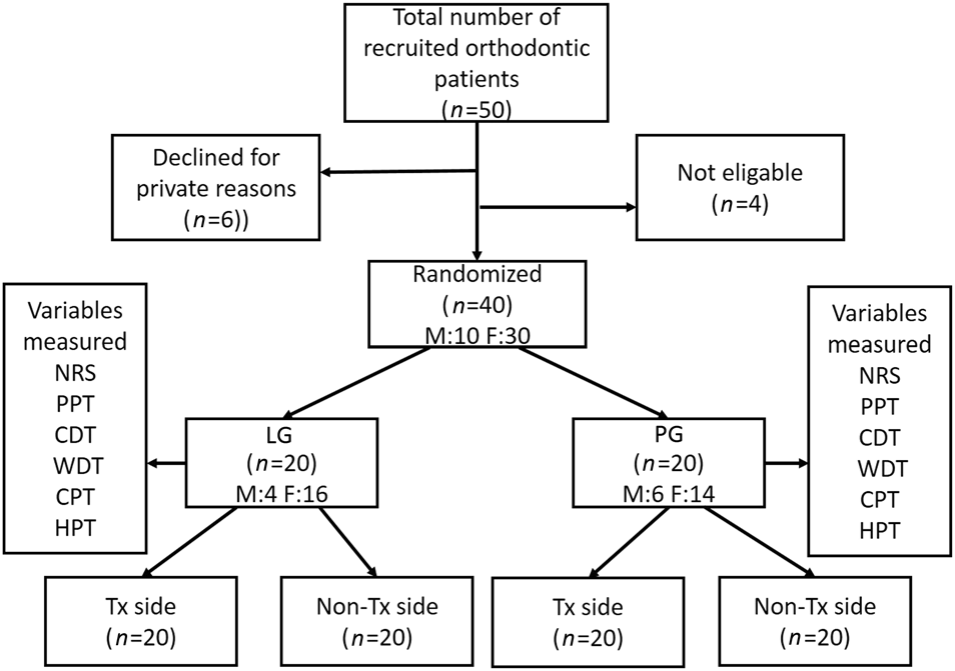
Flow diagram illustrating the study protocol and recruitment of the participants. Tx: treatment side. The variables measured were the NRS score, PPT, CDT, WDT, CPT, and HPT

The mean NRS pain score was 1.0 ± 1.6 in the LG and 2.1 ± 2.3 in the PG (ANOVA; *F* = 5.57, df = 1, *P* < 0.001). There was also a significant effect of time (ANOVA; *F* = 24, df = 4, *P* < 0.001), with the highest NRS scores being recorded at 24 h (*P* = 0.018). However, there was no significant interaction between the groups and time (ANOVA; *F* = 1.5, df = 4, *P* = 0.234) (Fig. 1).

### PPT at the canine tooth

**Fig. 2:**
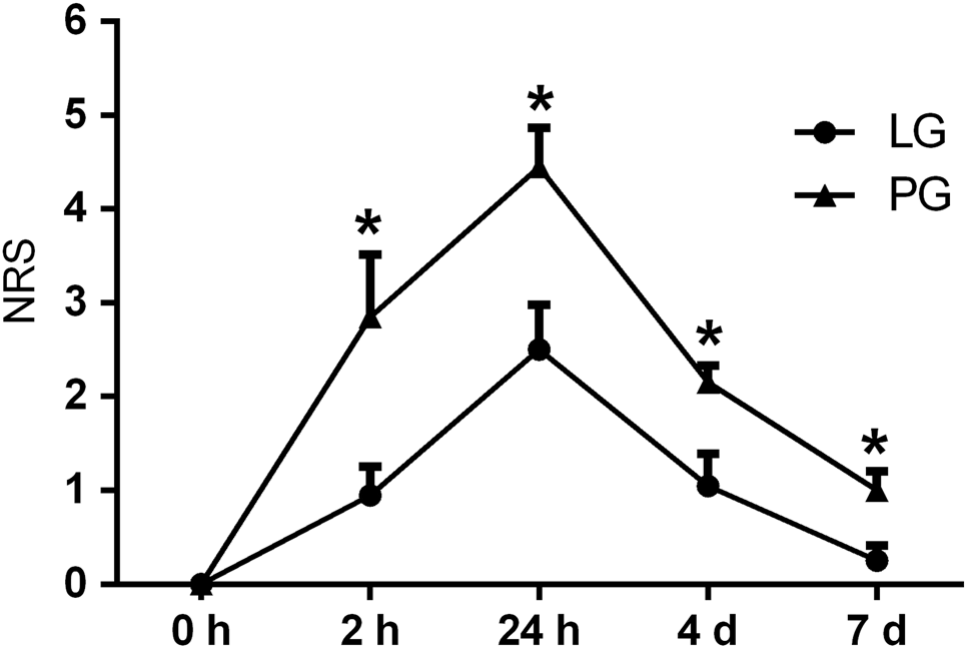
Pain intensities reported on the 0–10 NRS at 0 h, 2 h, 24 h, 4 d, and 7 d in the laser group (LG, *n* = 20) and placebo group (PG, *n* = 20). The data are presented as the mean + standard error of the mean. *indicates a significant difference between the groups (*P* < 0.05)

The mean PPT values at the canine teeth are shown in Table 1. There were significant effects of group (ANOVA; *F* = 54, df = 1, *P* < 0.013), side (ANOVA; *F* = 8.7, df = 1, *P* = 0.004) and time (ANOVA; *F* = 16.1, df = 1, *P* < 0.001) and a significant interaction between group, side, and time (ANOVA; *F* = 2.5, df = 4, *P* = 0.046). Moreover, the interaction between group and time was also significant (ANOVA; *F* = 12.5, df = 4, *P* < 0.001); whereas, the interaction between side and time was not significant (ANOVA; *F* = 2.3, df = 4, *P* = 0.711). The relative PPT changes were significantly larger in the LG compared with the PG at 2 h (ANOVA; F = 54.1, df = 1, *P* < 0.001), 24 h (ANOVA; *F* = 33.7, df = 1, *P* < 0.001), 4 d (ANOVA; *F* = 27.8, df = 1, *P* < 0.001), and 7 d (ANOVA; *F* = 24.8, df = 1, *P* < 0.001) at the treatment side and non-treatment side. When the treatment side in the LG was compared to the non-treatment side in the same group, there were significant effects of side (ANOVA; *F* = 10, df = 1, *P* < 0.001) and time (ANOVA; *F* = 5, df = 4, *P* < 0.001) but no significant interaction between side and time (ANOVA; *F* = 3.8, df = 4, *P* = 0.064). The relative PPT changes on the treatment side were significantly higher than those on the non-treatment side from 2 h to 7 days in the LG (*P* < 0.001, Fig. 2a). In the PG, there were no differences between the sides (*P* = 0.361, Fig. 2a).

**Table 1:** Average QST values across all of the time points on the treatment (Tx) and non-treatment sides in the LG and PG (means ± SD)

Items	CDT/°C	WDT/°C	CPT/°C	HPT/°C	PPT (gingiva)/kPa	PPT (tooth)/kPa
*Treatment side*						
LG	25.1 ± 4.8	45.6 ± 1.9	14.1 ± 5.2	47.2 ± 1.9	97.3 ± 42.2	279.2 ± 114.1
PG	27.4 ± 4.1	43.4 ± 2.2	19.4 ± 6.2	47.6 ± 1.9	84.1 ± 45.8	213.5 ± 123.7
*P*1 (LG vs PG)	0.061	0.11	<0.001^a^	<0.01^a^	0.512	<0.013^a^
*Non-treatment side*						
LG	23.4 ± 4.4	45.3 ± 2.2	16.5 ± 4.9	47.2 ± 1.7	92.2 ± 41.4	235.3 ± 111.2
PG	28.2 ± 4.3	43.2 ± 2.6	19.2 ± 6.8	47.4 ± 1.9	96.4 ± 49.4	212.2 ± 121.9
*P*2 (LG vs PG)	0.023^a^	0.043^a^	<0.001^a^	0.401	0.033^a^	<0.01^a^
*LG vs PG*						
*P*3 (LG vs PG)	0.003^a^	0.009^a^	<0.001^a^	0.009^a^	0.172	<0.001^a^

*CDT* cold detection threshold, *WDT* warmth detection threshold, *CPT* cold pain threshold, *HPT* heat pain threshold, *PPT* pressure pain threshold

*P*1 = relative changes of the Tx side between the groups

*P*2 = relative changes of the non-Tx side between the groups

*P*3 = relative changes between the two groups (including both the Tx and non-Tx sides)

^a^Indicates significant changes between the groups

Interestingly, the relative PPT changes at the non-treatment side in the LG were significantly higher compared with those at the non-treatment side in the PG at 2 h (ANOVA; *F* = 15, df = 1, *P* < 0.001), 24 h (ANOVA; *F* = 7.9, df = 1, *P* < 0.001), 4d (ANOVA; *F* = 7.39, df = 1, *P* = 0.01), and 7d (ANOVA; *F* = 7.65, df = 1, *P* < 0.001). Finally, there was a significant interaction between group and time (ANOVA; *F* = 4.8, df = 4, *P* < 0.001).

### PPT at gingiva

The mean PPT values at the gingiva are shown in Table 1. There were no significant effects of group (*P* = 0.172) and side (*P* = 0.243) but a significant effect of time (*P* < 0.001). However, when the non-treatment sides in the LG and PG were compared, there were significant effects of group (ANOVA; *F* = 5, df = 1, *P* = 0.033) and time (ANOVA; *F* = 11, df = 4, *P* < 0.001). The interaction between group and time was not significant (ANOVA; *F* = 1.29, df = 4, *P* = 0.290) (Fig. 2b).

### Thermal testing at the gingiva

The mean thermal QST values are shown in Table 1.

#### CDT

**Fig. 3:**
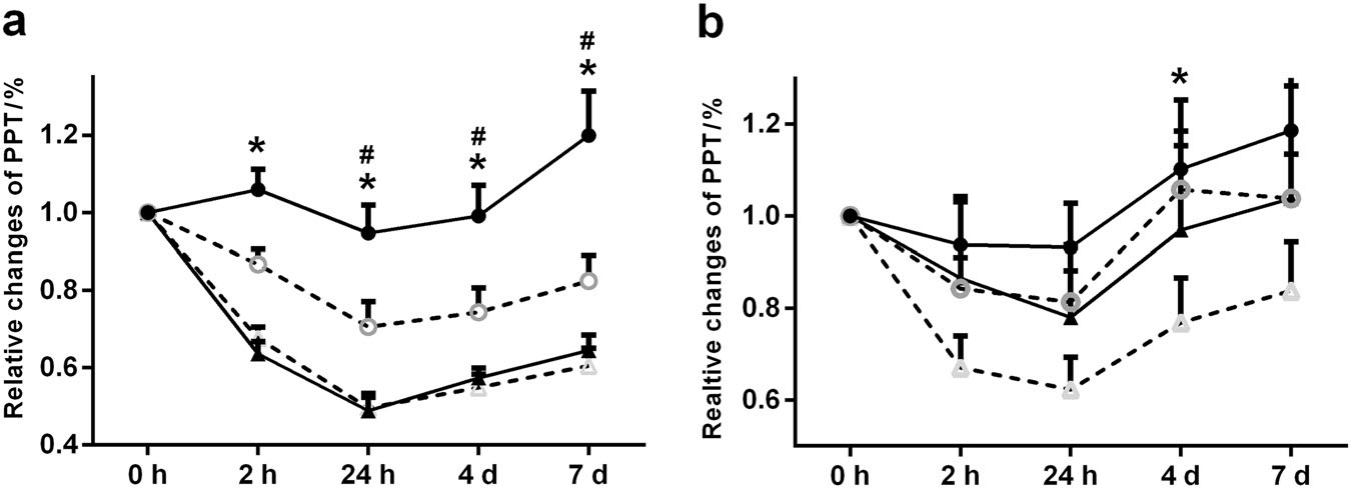
PPTs at **a** the canine tooth and **b** gingiva at 0 h, 2 h, 24 h, 4 d, and 7 d in the laser group (LG, *n* = 20) and placebo group (PG, *n* = 20). The data are presented as the mean ± standard error of the mean. Black circles represent LG- treatment side; white circles represent LG- non-treatment side; black triangles represent PG- treatment side; white triangles represent PG- non-treatment side. *Indicates a significant difference (*P* < 0.05) between the treatment groups; #indicates a significant difference between the treatment side and non-treatment side

There were significant effects of group (ANOVA; *F* = 9.2, df = 1, *P* = 0.003) and time (ANOVA; *F* = 14, df = 4, *P* < 0.001) but no significant effect of side (ANOVA; *F* = 0.3, df = 1, *P* = 0.565). The interaction between group, side and time was not significant (ANOVA; *F* = 0.36, df = 4, *P* = 0.834), but the interaction between group and time was significant (ANOVA; *F* = 4.3, df = 4, *P* = 0.003). The relative CDT changes were significantly smaller in the LG than in the PG at 2 h (ANOVA; *F* = 15, df = 1, *P* < 0.001), 24 h (ANOVA; *F* = 7.9, df = 1, *P* < 0.001), 4 d (ANOVA; *F* = 7.39, df = 1, *P* = 0.010), and 7 d (ANOVA; *F* = 7.65, df = 1, *P* < 0.001) (Fig. 3a). The relative CDT changes on the non-treatment side were also significantly smaller in the LG than in the PG from 24 h to 7 d (*P* = 0.023, Fig. 3a).

#### WDT

The mean WDT values are shown in Table 1.There were significant effects of group (ANOVA; *F* = 7.2, df = 1, *P* = 0.009) and time (ANOVA; *F* = 6.9, df = 4, *P* < 0.001) but no significant effect of side (ANOVA; *F* = 0.8, df = 1, *P* = 0.377). The interaction between group, side and time was not significant (ANOVA; *F* = 1.16, df = 4, *P* = 0.336), but the interaction between group and time was significant (ANOVA; *F* = 2.5, df = 4, *P* = 0.050). The relative WDT changes were significantly smaller in the LG than in the PG at 2 h (ANOVA; *F* = 5.8, df = 1, *P* = 0.021), 24 h (ANOVA; *F* = 5.7, df = 1, *P* = 0.023), and 4 d (ANOVA; *F* = 8.6, df = 1, *P* < 0.001) (Fig. 3b). The relative WDT changes on the non-treatment side were also significantly smaller in the LG than in the PG from day 4 to 7 (*P* = 0.043, Fig. 3b).

#### CPT

The mean CPT values are shown in Table 1. There were significant effects of group (ANOVA; *F* = 28, df = 1, *P* < 0.001) and time (ANOVA; *F* = 9.1, df = 4, *P* < 0.001) but no significant effect of side (ANOVA; *F* = 2.5, df = 1, *P* = 0.117). The interaction between group, side, and time was not significant (ANOVA; *F* = 0.17, df = 4, *P* = 0.953), but the interaction between group and time was significant (ANOVA; *F* = 7.6, df = 4, *P* = 0.050). The relative CPT changes were significantly smaller in the LG than in the PG at 2 h (ANOVA; *F* = 26.3, df = 1, *P* < 0.001), 24 h (ANOVA; *F* = 22.2, df = 1, *P* < 0.001), 4 d (ANOVA; *F* = 19.1, df = 1, *P* < 0.001), and 7 d (ANOVA; *F* = 18.4, df = 1, *P* < 0.001) (Fig. 3b). The relative CPT changes were significantly smaller on the treatment side than on the non-treatment side in the LG from 2 h to 7 d (*P* < 0.001). The relative CPT changes were also significantly lower on the non-treatment side than on the treatment side in the PG at 2 h, 24 h, 4 d and 7 d (ANOVA; *F* = 8.2, df = 1, *P* < 0.001) (Fig. 3c).

#### HPT

The mean HPT values are shown in Table 1. There were significant effects of group (ANOVA; *F* = 7.1, df = 1, *P* = 0.009) and time (ANOVA; *F* = 5.1, df = 4, *P* < 0.001) but no significant effect of side (ANOVA; *F* = 0.9, df = 1, *P* = 0.358). The interaction between group, side and time was not significant (ANOVA; *F* = 1.1, df = 4, *P* = 0.390). The relative HPT changes were significantly larger in the LG than in the PG from 24 h to 7 d (Fig. 3d).

### QST at the hand

There were no significant differences between the LG and PG for the CDT (ANOVA; *F* = 0.005, df = 1, *P* = 0.945), WDT (ANOVA; *F* = 0.33, df = 1, *P* = 0.572), CPT (ANOVA; *F* = 0.03, df = 1, *P* = 0.864), HPT (ANOVA; *F* = 0.001, df = 1, *P* = 0.976) and PPT (ANOVA; *F* = 1.25, df = 1, *P* = 0.274). There were significant time effects for CDT (ANOVA; *F* = 5.63, df = 4, *P* < 0.001), WDT (ANOVA; *F* = 3.3, df = 4, *P* = 0.021), CPT (ANOVA; *F* = 2.7, df = 4, *P* = 0.045), and HPT (ANOVA; *F* = 3.8, df = 4, *P* = 0.011), but there was no time effect for PPT (ANOVA; *F* = 1.6, df = 4, *P* = 0.191) (Table 2).

**Table 2:** Mean QST values across all of the time points on the hand in the laser group (LG, *n* = 20) and placebo group (PG, *n* = 20)

Items	CDT/°C	WDT/°C	CPT/°C	HPT/°C	PPT/kPa
LG	28.4 ± 2.5	34.7 ± 2.4	21.7 ± 5.1	40.6 ± 3.2	247.9 ± 109.1
PG	27.7 ± 3.4	35.6 ± 2.1	20.5 ± 6.6	41.4 ± 2.8	247.7 ± 77.4
*P* (LG vs PG)	0.945	0.572	0.864	0.976	0.274

*CDT* cold detection threshold, *WDT* warmth detection threshold, *CPT* cold pain threshold, *HPT* heat pain threshold, *PPT* pressure pain threshold

*P* = relative changes between the two groups

## Discussion

The effect of the repeated application of LLLT on orthodontic pain was evaluated in the present study. The self-reported intensity of orthodontic pain and mechanical sensitization were significantly reduced by LLLT compared with the placebo treatment. Interestingly, the degree of thermal sensitization of the gingiva evoked by the orthodontic treatment was also significantly decreased by LLLT. Surprisingly, the LLLT treatment affected not only the treatment side but also the non-treatment side, indicating that there is a generalized effect within the trigeminal system. However, there were no effects of the LLLT on extra-trigeminal sensitivity.

### LLLT effect on orthodontic pain

The laser used in this study was an 810-nm gallium-aluminium-arsenic diode laser in continuous mode with the power set at 400 mW. The reason for choosing this spectrum was to obtain more tissue penetration and biostimulation.^19^ It has been shown that similar laser stimulation^35^ can significantly inhibit the production of IL-1 beta and prostaglandin E2.^35^ The use of LLLT to reduce orthodontic pain has, indeed, been attempted for many years.^16,28,36,37^ Significant decreases in self-reported pain scores with LLLT after the placement of the initial arch wires have been shown in a double-blinded study.^23^ Similar findings were also reported in another study,^17^ in which significant pain reduction was observed with LLLT immediately after the insertion of separators until day 4.

The participants in the present study reported maximum levels of pain at 24 h after the arch wire was activated in both the PG and LG. Then, the self-reported pain decreased slowly and disappeared by approximately day 7. This agrees with the results of studies that reported maximum pain after 24 h of the application of orthodontic forces.^37^ The NRS was used as a pain measurement tool in this study, and lower NRS pain scores were observed in the LG compared with the PG from 2 h to 7 d (Fig. 1). A double-blinded study evaluated orthodontic pain every day for 7 d after the placement of the first orthodontic arch wire and reported significant decreases in the self-reported pain intensity with LLLT on the most painful day.^21^ In addition, the duration of orthodontic pain also appears to be shorter following LLLT.^21^ The findings in the present study are consistent with these previous reports on the analgesic effects of LLLT on orthodontic pain.

The exact mechanisms responsible for the apparent analgesic effect of LLLT are still unclear.^14^ It is assumed that perhaps LLLT has anti-inflammatory and neural regenerative properties as a probable result of the biological reactions that stimulate cell differentiation and proliferation, enabling it to have antinociceptive effects.^22,37,38^ Animal studies have shown that LLLT could achieve a reduction of inflammatory processes similar to those shown from the use of non-steroidal, anti-inflammatory drugs.^39^ Furthermore, LLLT might improve the blood supply and enhance tissue recovery,^37,39^ and LLLT might play an important role in improving the healing procedures of soft tissue.^40^ Other factors contributing to the analgesic effect of LLLT might be the reactivation of enzymes targeted at nociceptive promoting factors, inhibiting nerve depolarization (C fibers in particular), ATP production, and prostaglandin reduction.^14,41^ In addition, LLLT might alter nerve conduction by influencing the synthesis, release, and metabolism of encephalin, endorphins and many other neurochemicals.^14,42^ This study was not designed to elucidate the neurobiological mechanisms potentially underlying an analgesic effect of LLLT on orthodontic pain, but the present findings so far indicate that the observed reduction in the NRS pain scores cannot be attributed to placebo-based mechanisms. Although placebo effects cannot be completely ruled out based on the self-reported NRS pain scores, the observation of consistent and significant effects on somatosensory function further supports the notion of a neurobiological effect of LLLT.

### LLLT effect on somatosensory function

As a special feature of the present study, intra-oral QST was applied in addition to traditional self-reports of pain intensity evoked by the orthodontic treatment. QST is believed to contribute to a better understanding and profiling of the underlying neurobiological mechanisms related to orthodontic pain.^33^ To our knowledge, it was the first time that QST was applied to evaluate the effect of LLLT on orthodontic pain in a systematic manner. The QST parameters are considered to offer a diagnostic sensitivity of 67%–100% for small-fiber neuropathy and to be potentially valuable for evaluating small-fiber function.^43,44^ The CDT is considered to represent small myelinated Aδ nerve fiber conduction, the WDT is considered to represent unmyelinated C nerve fiber conduction, and the CPT and HPT may represent conduction by both Aδ and C nerve fibers.^45,46^ The PPT was measured to test deep pain sensitivity, which is probably mediated through both C and Aδ fibers.^47^ The PPT can also be used to assess mechanical pain sensitivity in the periodontal ligament and gingiva.^32^

The present QST results demonstrated that not only tooth-related pain but also sensitization of thermal and mechanical somatosensory channels were evoked by the orthodontic treatment. In fact, both mechanical (PPT) sensitization and thermal (CDT, WDT, CPT, and HPT) sensitization were observed during the orthodontic treatment until day 7. In the present study, all of the QST parameters were normalized to their baseline values (0 h), and the relative changes at the 5 time points in the two treatment groups were calculated and presented (Figs. 2 and 3). Interestingly, smaller relative changes (decreased sensitivity for all of the parameters) were observed in the LG group compared with the PG group throughout the treatment. It can be speculated that LLLT may have decreased peripheral sensitization of Aδ fibers and C afferent nerve fibers, which could be responsible for the changes in the QST parameters in the LG observed in the present study.

Mechanical allodynia in the periodontal ligament around the canine tooth was investigated with the same method used in our previous study.^32^ The force from the pressure algometer was applied directly to the crown of the canine tooth, and the recorded PPT may partially reflect the innervation patterns and receptor density in the periodontal ligament.^48^ Interestingly, in the present study, significantly higher PPTs (i.e., decreased sensitivity) on the LLLT-treated side compared to the non-treated side were observed in the LG. Thus, it can be speculated that LLLT may decrease the sensitization of the periodontal ligament evoked by orthodontic treatment. Surprisingly, the analgesic effect induced by LLLT was not restricted to the treatment side and was also observed on the contralateral side, indicating an extended effect in the trigeminal area. Studies on animals have shown the existence of branched nerves innervating both intrapulpal and periodontal tissues.^49^ It has also been shown in animal studies that LLLT may promote neural regeneration,^37^ but until now, only few animal studies have been available that allow for the study of the activation of the trigeminal-vascular system. Within the meninges, the activation of the trigeminal-vascular system (electrically or chemically) leads to an ipsilateral inflammatory response characterized by vasodilation.^50^ Animal studies have also indicated that noxious stimulation may produce marked blood flow changes in various orofacial structures.^51^ Noxious stimulation of the human teeth is indeed associated with bilateral increases in blood flow in both the maxillary and mandibular nerve innervation territories.^52^ In the trigeminal-vascular system in rats, prostaglandin E2 (PGE2) is considered to be a key mediator for pain and nocifensive responses.^53^ PGE2 causes vasoconstriction and is increased in both blood and saliva during migraine attacks, which is a mechanism related to pain. Interestingly, PGE2 may be reduced by LLLT,^13^ which is related to the activation of the trigeminal-vascular system, which, again, might be associated with the bilateral effects of LLLT.

Tooth pain caused by orthodontic tooth movement is associated with elevated gingival crevice fluid contents of matrix metalloproteinase (MMP)-8 and neuropeptide substance P (SP) levels, which are more abundant in pulp tissues from painful human teeth^54^ and lead to an increase in the intrapulpal pressure.^55^ Thus, increased fluid flow from the root canal system through the apical foramen and dentinal tubules may cause the delivery of SP and MMP-8 to the periodontal ligament space.^56^ These data support the possibility for the local neurogenic spread of inflammation from intrapulpal tissues to surrounding periodontal tissues, which may further explain the bilateral effect of LLLT.

Pain only evoked local elevations in the gingival crevice fluid content of SP and MMP-8 levels and caused no marked modulations in systemic cardiovascular parameters. Thus, systemic stress mechanisms probably did not significantly contribute to the present results, which may explain the lack of significant QST effects on the hand. Time effects of the QST parameters at control sites, such as the hand, have also been observed in a previous study and may be attributed to minor drifts in psychophysical performance, e.g., an adaptation or a habituation of the test procedures.^33^

The process of bone remodeling in periodontal tissues is a major determinant of orthodontic tooth movement.^57^ The evidence suggests that LLLT can be effective in enhancing the rate of orthodontic tooth movement since LLLT is found to increase the rate of bone remodeling without imposing any adverse effects.^58,59^ However, other studies have reported conflicting results^17,60^ and have failed to observe any significant improvement in the rate of orthodontic tooth movement associated with LLLT.^61^ The rate of tooth movement was not recorded in the present study, and further studies will be needed to clarify the effect that LLLT has on tooth movements.

### Limitations of the study

One of the limitations of the present study could be argued to be the mixed group of participants, which includes both children and adults and includes participants of both genders. A total of 10 individuals were under 18 years old, but so far, there is no systematic information about age-related differences in acute pain responses evoked by orthodontic treatment, although gender differences in pain sensitivity have been documented.^62^ Nevertheless, the strength of the mixed study group is that the present results may be more generalizable. Another limitation of the present study could be argued to be the subjective nature of the self-reported pain. However, in addition to self-reports of pain, we also included psychophysical responses (QST), which also indicated significant effects on the treatment side in the LG. In future studies, objective measures of somatosensory function (e.g., blink reflexes) and nociceptive activity (e.g., biomarkers from the gingiva, such as prostaglandins and other pain mediators) could be incorporated into the study design to further support the potential analgesic effect of LLLT in relation to orthodontic pain. A particular strength of the present study was its double-blinded and placebo-controlled design. However, a limitation may still be that no information was collected to demonstrate that participants were indeed successfully blinded and that participants in the PG had the same degree of anticipation and expectancy of a positive outcome. Previous studies have clearly demonstrated that expectancy reports are crucial for the understanding of placebo effects.^63^ Thus, it may not be possible to completely rule out that the present findings were not contaminated by placebo effects. However, the QST findings may nevertheless suggest that there could be physiological responses related only to LLLT. Further studies are obviously needed to address these questions in more detail.

## Materials and methods

### Participants

The volunteers were recruited from the orthodontic clinic at the Hospital of Stomatology of Nanjing Medical University (Nanjing, China). The inclusion criteria were individuals who were going to start comprehensive orthodontic treatment for slight crowding without tooth extraction, and all of the participants were to be healthy without signs or symptoms of pain or any kinds of on-going therapy and no other systemic disease. The exclusion criteria were active caries, periodontal diseases, visible lesions of the oral mucosa, and any kind of chronic use of analgesics or drugs affecting the function of the central nervous system.

**Fig. 4:**
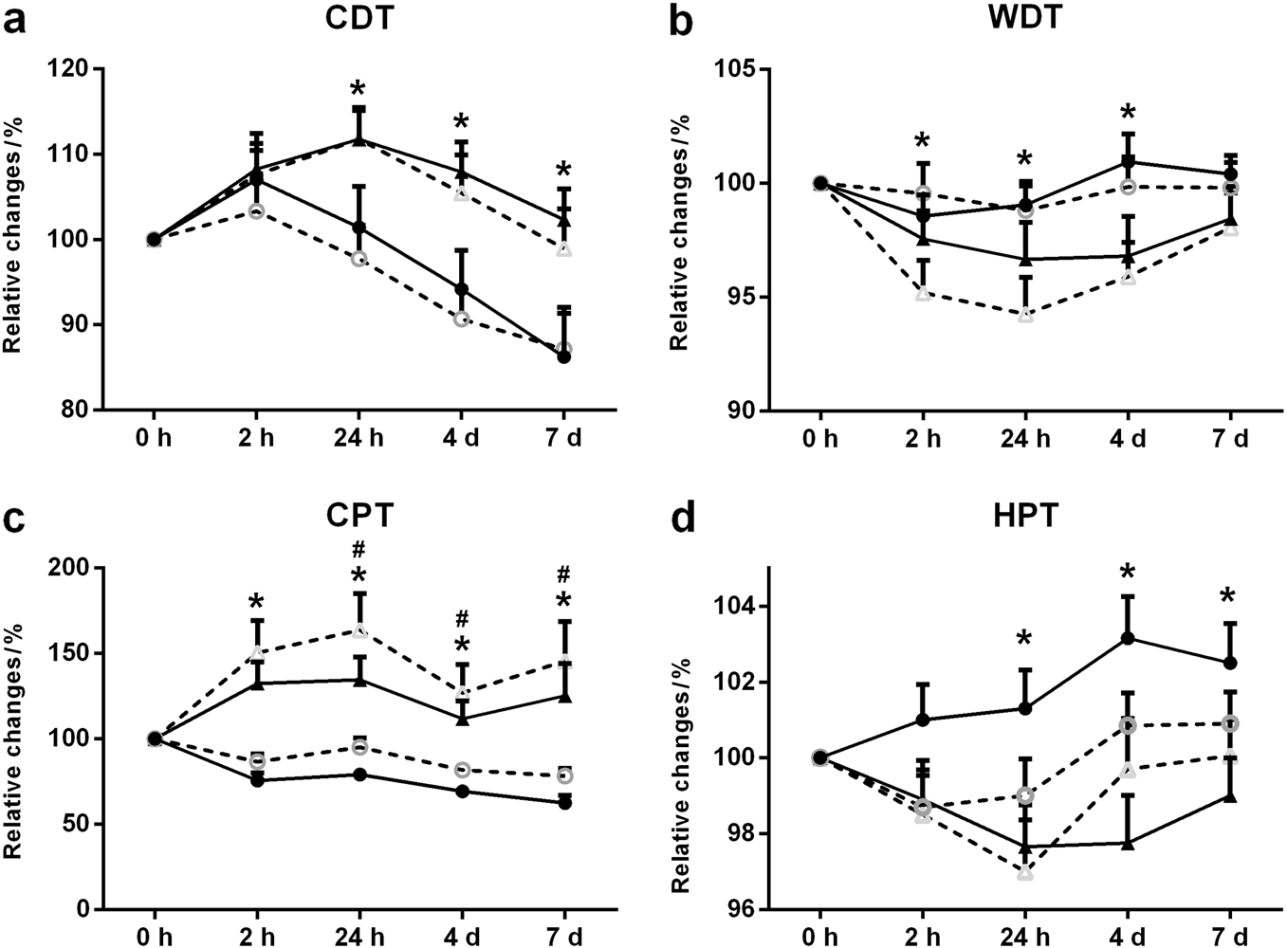
Relative changes for CDT, WDT, CPT, and HPT of the participants. **a** CDT, **b** WDT, **c** CPT, and **d** HPT at 0 h, 2 h, 24 h, 4 d, and 7 d in the laser group (LG, *n* = 20) and placebo group (PG, *n* = 20). The data are presented as the mean ± standard error of the mean. Black circles represent LG- treatment side; white circles represent LG- non-treatment side; black triangles represent PG- treatment side; white triangles represent PG- non-treatment side. *indicates a significant difference (*P* < 0.05) between the groups; #indicates a significant difference between the treatment side and non-treatment side

Fifty participants requiring orthodontic treatment were initially recruited in this study: six participants declined enrollment after receiving study information, and an additional four individuals were excluded according to the exclusion criteria. A total of 40 participants (10 males and 30 females; 12–33 years old, mean ± SD: 20.8 ± 5.9 years) were included in the study (Fig. 4). Ten individuals were under 18 years old (10–15 years old), but additional informed consent was obtained from their parents. Only those children who could cooperate and understand the instructions and study information were included.^17,19,20^ All of the participants gave informed consent to the procedures, which were approved by the local Ethical Committee (approval number: PJ2016-031-001) in accordance with the Helsinki Declaration II. In addition, all of the participants understood that they were free to withdraw from the experiment at any time.

### Study design

The experiment was performed as a randomized, placebo-controlled and double-blinded trial. All of the participants were tested by the same examiner (Examiner 1: Song Wu). The laser treatment and randomization were performed by another examiner (Examiner 2: Huijie Shen). The forty participants were randomly divided into two experimental groups: a LG and a PG. The randomization code of the two treatment groups (20 in each group) for the 40 participants was obtained using a computer program (www.randomizer.org) by Examiner 2. The LG received the low-level laser therapy (LLLT) on one randomized side from Examiner 2 immediately after (0 h) the arch wire (0.014 super plastic nickel-titanium arch wire) was placed in self-ligating brackets (American Orthodontics, Sheboygan, WI, USA) and then again at 2 h, 24 h, 4 d, and 7 d. The PG received inactive treatment with the LLLT device, which was applied in a similar way at the same test sites and at the same time points as for the LG. The treatment sides in the LG and PG groups were also randomly determined using the same computer program by Examiner 2. QST was performed after the application of active LLLT or placebo LLLT in both of the groups. A 0–10 numerical rating scale (NRS) score, in which 0 represented “no pain” and 10 indicated “the most pain imaginable”, was recorded from the participants, and the modified intra-oral QST was performed at the canine tooth and the surrounding gingiva on both sides at 0 h, 2 h, 24 h, 4 d, and 7 d after the orthodontic forces were applied. The participants were requested to close their eyes during the LLLT application. The study was performed in a quiet and temperature-controlled room. The contralateral, non-treatment side served as an additional control in both groups. Examiner 1 was blinded to the information about the allocation to the LG or PG, as well as to which side had been treated or not.

### Intra-oral QST

#### Thermal testing

Thermal quantitative sensory tests were performed using a computerized thermal stimulator (MEDOC TSA-2001 apparatus, Medoc Ltd, Ramat-Yishai, Israel). The contact area of the intra-oral thermode was 6 mm× 6 mm. The cold detection threshold (CDT), warmth detection threshold (WDT), cold pain threshold (CPT) and heat pain threshold (HPT) were tested at the area of the gingiva around the upper-left and right canines and on the left hand (as an extra-segmental control) at the five time points. The thermode started from a baseline temperature of 37 °C. The temperature decreased or increased by 1 °C·s^-1^. To determine the CDT, WDT, CPT, and HPT, the participants were asked to press a computer mouse button as soon as they perceived the slightest sensation of cold or warmth (CDT and WDT) or when the temperature reached a sensation of painful hot or cold (CPT and HPT). The temperature of the thermode returned to the baseline after the mouse button had been pushed. The range of the stimulation temperatures was between 0 °C and 50 °C. The CDT and the WDT were always measured first, followed by the CPT and the HPT. The mean of three repeated trials was used to determine the threshold values.

#### Mechanical testing

The mechanical PPT was measured by an algometer (Medoc, Ramat-Yishai, Israel). The diameter of the intra-oral probe was 8 mm. The algometer was applied perpendicular to the surface of the test sites. The measuring sequence was the left hand, upper-left canine gingiva, upper-right canine gingiva, upper-left canine, and upper-right canine.^33^ The pressure was increased with a constant application rate of 30 kPa·s^-1^. The participants were instructed to concentrate on the test stimulus and to press the switch button as soon as they felt that the pressure changed to the slightest sensation of pain. The amount of pressure (kPa) at this point was defined as the PPT. Three measurements per site were made at 1-min intervals to obtain a mean value.

### Low-level laser therapy

LLLT was applied buccally and lingually to an upper canine at 6 points: mesial, distal, and at a site corresponding to the middle of the root of the canine tooth for 20 s each in the LG. The laser tip diameter was 1 mm, and the laser tip was kept 10 mm away from the surface of the gingiva during the stimulation. From the cementoenamel junction of the tooth towards the apex of the tooth, the laser tip was directed perpendicular to the long axis of the tooth. In the PG, the LLLT device was held at the same 6 points for the same duration with the light on for the operation indicator. The same procedure was repeated for the LG but without any active laser output. Both the examiner and the participant wore protective laser glasses. LLLT was applied using an 810-nm gallium-aluminium-arsenic diode laser in continuous mode with the power set at 400 mW, 2 J·cm^-2^ (Pilot, Germany).

### Statistical analysis

The sample size was calculated with risks for type I and type II errors of 5% and 20%, respectively, an estimate of the inter-individual variation of 25% and a minimal relevant difference for detection of 20%. A total of 40 patients were recruited in the present study.

Descriptive statistics were used to summarize the data. Baseline comparisons between the two groups in regards to age, gender and the QST parameters at baseline were performed. The necessary logarithmic transformation was performed when the data were not normally distributed. The mean values and SD of the NRS score, CDT, WDT, CPT, HPT, and PPT at each time point and test site were calculated. All of the data were normalized to the baseline, and the relative changes of each parameter were compared between the treatment groups. A three-way mixed model analysis of variance (ANOVA) with repeated measures was used to analyse the different outcome parameters of the CDT, WDT, CPT, HPT, and PPT at the test sites at different time points. The between group factor in the ANOVA was group (LG and PG), while the within-group factors were side (treatment and non-treatment side), and time point (5 levels: 0 h, 2 h, 24 h, 4 d and 7 d). Post hoc tests were performed with the LSD honestly significant difference test with corrections for multiple comparisons. The significance level was set at 0.05. Blinding of the data was maintained in the statistical analysis.

### Data availability

The QST data that support the findings of this study are available in figshare with the identifier 10.6084/m9.figshare.5532226.v1.

## Conclusion

A repeated application of LLLT using a gallium-aluminium-arsenic diode laser with an 810-nm wavelength was able to significantly reduce self-reported pain scores and sensitization of the periodontal and gingival tissues evoked by orthodontic treatment.

The effect also extended to the contralateral side in the trigeminal region but not in the extra-trigeminal region, indicating that LLLT treatment may have some degree of bilateral effects within the orofacial region. This interesting finding calls for future studies on the clinical application of LLLT with larger cohorts, as well as for those including additional measures of nociceptive function and sensitization.
